# Predicting Agenesis of the Mandibular Second Premolar from Adjacent Teeth

**DOI:** 10.1371/journal.pone.0144180

**Published:** 2015-12-16

**Authors:** Geetanjali Sharma, Ama S. Johal, Helen M. Liversidge

**Affiliations:** Queen Mary University of London, Barts and the London School of Medicine and Dentistry Institute of Dentistry, London, United Kingdom; University of Otago, NEW ZEALAND

## Abstract

Early diagnosis of agenesis of the mandibular second premolar (P2) enhances management of the dental arch in the growing child. The aim of this study was to explore the relationship in the development of the mandibular first molar (M1) and first premolar (P1) at early stages of P2 (second premolar). Specifically, we ask if the likelihood of P2 agenesis can be predicted from adjacent developing teeth. We selected archived dental panoramic radiographs with P2 at crown formation stages (N = 212) and calculated the likelihood of P2 at initial mineralisation stage ‘Ci’ given the tooth stage of adjacent teeth. Our results show that the probability of observing mandibular P2 at initial mineralisation stage ‘Ci’ decreased as both the adjacent P1 and M1 matured. The modal stage at P2 ‘Ci’ was P1 ‘Coc’ (cusp outline complete) and M1 ‘Crc’ (crown complete). Initial mineralisation of P2 was observed up to P1 ‘Crc’ and M1 stage ‘R½’ (root half). The chance of observing P2 at least ‘Coc’ (coalescence of cusps) was considerably greater prior to these threshold stages compared to later stages of P1 and M1. These findings suggest that P2 is highly unlikely to develop if P1 is beyond ‘Crc’ and M1 is beyond ‘R½’.

## Introduction

An understanding of dental development is crucial for clinicians, especially those treating children and young adults. Recognising the normal timing of developing teeth during growth is fundamental. Distinguishing between late development and agenesis of a tooth can impact significantly on treatment choice. Agenesis of teeth affects 4–6% of the population with mandibular second premolars reported to be the most commonly missing teeth, after third molars [1,2]. Importantly, agenesis, with the exception of the third molar, frequently involves orthodontic treatment that is costly and complex, impacting on both public health funding bodies, the patients and their families. In some cases treatment can be confined to interceptive action provided an accurate and early diagnosis can be made [3]. Additional benefits of early diagnosis include financial, psycho-social and functional advantages.

The first evidence of cusp tip formation of the mandibular second premolar (P2) has been described as occurring during the second to fourth years [4,5]. Very late development of P2 has been reported in a few cases [6–8] but diagnosis of P2 agenesis is generally not confirmed prior to the age of six to nine years of age or even later (see Rakhshan [2]). A more biological approach is to express this threshold in terms of dental age rather than chronological age. One way to do this is to assess the developing teeth adjacent to the site of agenesis and to use this information to predicting the likelihood of agenesis. This approach was used to predict third molar agenesis from the development of the adjacent second molar and third molar crypt formation was not observed when the second molar was at stage root complete or later [9].

The aim of this study was to describe tooth development of the permanent mandibular first premolar (P1) and first molar (M1) at early radiographic stages of the permanent mandibular second premolar (P2). This information may contribute to assessing the likelihood of P2 formation in cases of late P2 formation or agenesis of P2. Such information can be gained from panoramic dental radiographs taken for diagnosis and treatment of routine dental care in young children.

## Materials and Methods

This was a retrospective study of archived panoramic radiographs taken at the Institute of Dentistry, Bart’s and the London School of Medicine and Dentistry, London. Ethical approval was obtained from Queen Mary Research Ethics Committee. Dental panoramic radiographs (N = 213) were selected from archived dental radiographs taken during diagnosis and treatment of local patients (White and Bangladeshi) attending the Institute of Dentistry. Age and sex of the sample is detailed in Table 1. The inclusion criteria for the selection of radiographs were good image quality, evidence of a mandibular second premolar (P2) crown formation (from initial cusp formation) and evidence of a developing mandibular first pre-molar and molar on the same side of the arch. Exclusions, were poor image quality or poor resolution in the P1, P2, M1 region and unusual pathology. Patient records/information was anonymized and de-identified prior to analysis.

**Table 1:** Age and sex of radiographic sample.

Age groups	male	female	total
2+	12	9	21
3+	48	44	92
4+	37	31	68
5+	1	5	6
6+	7	5	12
7+	3	2	5
8+	1	1	2
9+	0	3	3
unknown	3	1	4
Sum	112	101	213

2+ indicates children aged 2.00 to 2.99 years etc.

The first author recorded tooth formation stages of M1, P1 and P2 from panoramic radiographs. These teeth on the left side were examined without magnification using a standard light box. If this region of the radiograph was unclear on the left side, teeth on the right side were assessed. The modified description of Moorrees *et al*. [5] stages outlined in the Atlas of tooth development and eruption [10] (https://atlas.dentistry.qmul.ac.uk/) were used to identify tooth stages. At each stage, in addition to the criteria for that stage, the criteria for the previous stage were satisfied. In borderline cases the earlier stage was always assigned. Reliability of tooth stage assessment was done by repeat assessment of 14 radiographs after a period of two weeks. Cohen’s Kappa was calculated to measure the strength of agreement for intra-observer reliability.

Descriptive statistics of age (mean, standard deviation, minimum, maximum) were calculated for the sample, as well as individuals with P2 at stage ‘Ci’. Tooth stages of P1 and M1 were cross tabulated relative to P2 tooth stages. The earliest tooth stage of P2 at ‘Ci’ was of particular interest and the distribution of P1 and M1 stages conditional on P2 at ‘Ci’ was described (N = 89). Modal stage of development of P1 and M1 conditional on P2 at ‘Ci’ was described. The odds ratio (OR) of P2 being observed as stage ‘Cco’ (coalescence of cusps) or later was calculated indicating the chance of P1 up to ‘Crc’ stage divided by the chance of P1 root stages. This was done by dividing the data into four groups. The first group was the number of individuals with P1 up to and including stage ‘Crc’ and P2 at stage ‘Ci’. The second group included P1 stages later than ‘Crc’ and P2 at stage ‘Ci’. The third group was individuals with P1 stages up to and including ‘Crc’ and P2 stage ‘Cco’ and later. The fourth group included P1 stages later than ‘Crc’ and P2 stage ‘Cco’ and later. Similarly, the OR of P2 being observed as stage ‘Cco’ or later was calculated indicating the chance of M1 up to ‘R½’ stage divided by the chance of M1 ‘R¾’ or later root stages.

## Results and Discussion

### Reliability of stage assessment

Kappa value for intra-observer reliability of tooth stage assessment was 0.77 showing excellent agreement.

### Descriptive statistics

The sample of selected radiographs consisted of 112 males and 101 females with a mean age of 4.20 years (standard deviation 1.29, minimum 2.08, maximum 9.56) mostly made up of three and four year old children. Four children did not have recorded age. The mean age of P2 stage ‘Ci’ was 3.56 years (standard deviation 0.67, minimum 2.08, maximum 6.64, N = 87).

### Cross-tabulation of P1 and M1 relative to P2

Tooth stages were cross-tabulated to demonstrate the relationship of P2 development against M1 and P1 (Tables 2 and 3). M1 and P1 maturity is accompanied by an increase in P2 maturity. The M1 was more advanced in formation than P1, and P1 was more advanced than P2. The earliest stage of P2 ‘Ci’ was observed in 89 individuals and at this stage, P1 development ranged from ‘Cco’ to ‘Crc’ with the modal stage of P1 ‘Coc’. Only one individual at P2 ‘Ci’ was observed at P1 ‘Crc’ and none for P1 root stages in this study. Similarly, first molar stages at P2 ‘Ci’ were observed during late crown stages and early root stages of M1, with none being observed after root one half (when crown height = root length). The modal stage was M1 ‘Crc’. Stage P2 ‘Ci’ was observed at four to eight stages delayed relative to M1. These results show that the likelihood of P2 ‘Ci’ being observed after these stages is low and that the likelihood of P2 agenesis is high.

**Table 2:** Cross tabulation of P2 and P1 developmental stages.

P2 stage	P1 stage							
	Cco	Coc	C½	C¾	Crc	Ri	R¼	Total
Ci	21	35	23	9	1	0	0	89
Cco	0	12	22	28	1	0	0	63
Coc	1	1	12	20	2	0	0	36
C½	0	0	0	4	3	1	0	8
C¾	0	0	0	0	3	4	0	7
Crc	0	0	0	0	0	8	2	10
Total	22	48	57	61	10	13	2	213

Abbreviations: P1 (first premolar); P2 (second premolar); Ci (initial mineralisation); Cco (coalescence of cusps); Coc (cusp outline complete); C½ (crown half); C¾ (crown three quarters); Crc (crown complete); Ri (initial root); R¼ (root quarter).

**Table 3:** Cross tabulation of P2 and M1 developmental stages.

P2 stage	M1 stage						
	C¾	Crc	Ri	R¼	R½	R¾	Total
Ci	4	53	17	14	1	0	89
Cco	3	19	18	19	4	0	63
Coc	2	7	9	16	2	0	36
C½	1	0	0	4	0	3	8
C¾	0	0	0	0	1	6	7
Crc	0	0	0	0	0	10	10
Total	10	79	44	53	8	19	213

Abbreviations: M1 (first molar); P2 (second premolar); Ci (initial mineralisation); Cco (coalescence of cusps); Coc (cusp outline complete); C½ (crown half); C¾ (crown three quarters); Crc (crown complete); Ri (initial root); R¼ (root quarter); R½ (root half); R¾ (root three quarters).

**Fig 1:**
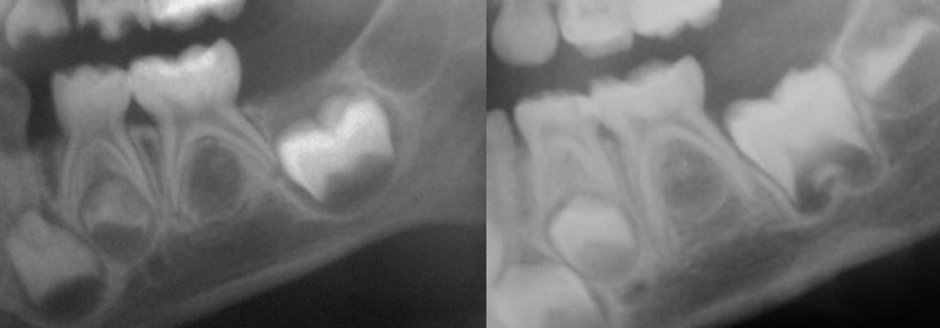
Radiographic example of modal stages of P1, P2 and M2 at P2 stage ‘Ci’ (left) and an individual with P2 stage ‘Ci’ relatively late (right). The modal stages were P1 ‘Coc’ and M1 ‘Crc’. In the case of late initiation, stages were P1’C3/4’ and M1 ‘R1/4’.

**Fig 2:**
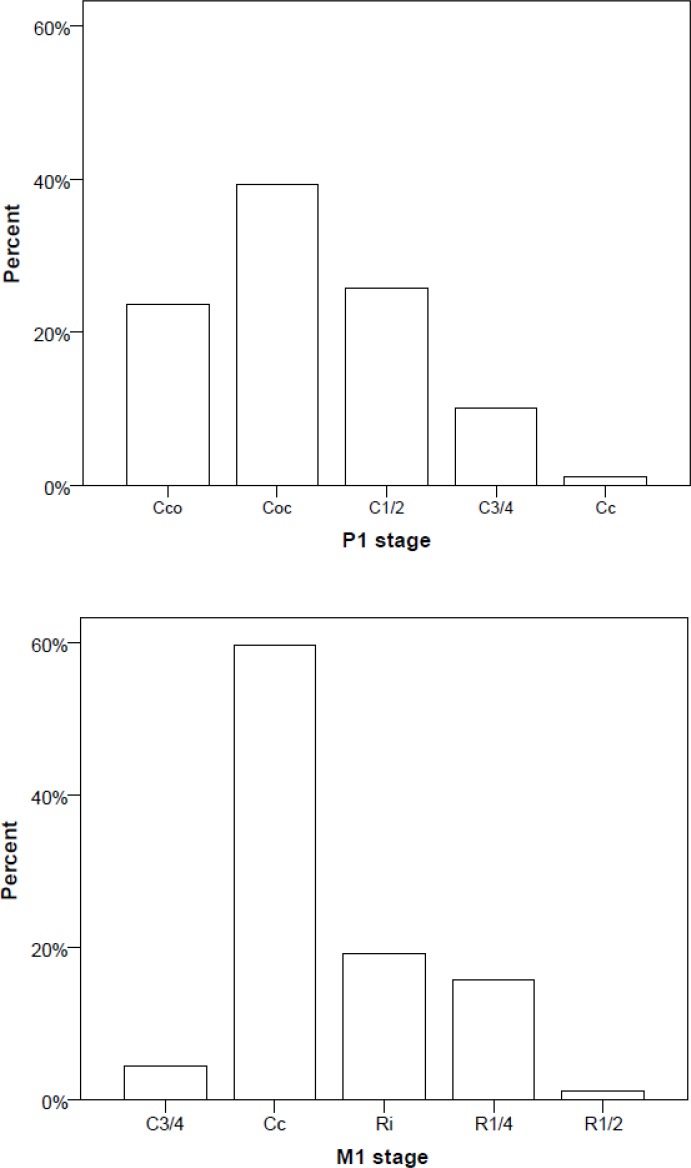
Distribution of P1 and M1 stages conditional on P2 at initial mineralisation stage ‘Ci’ (N = 89). P2 stage ‘Ci’ was not observed once P1 had reached ‘Ri’ or M1 ‘R3/4’.

A radiographic example of the modal stages of P1 ‘Coc’ and M1 ‘Crc’ conditional on P2 at ‘Ci’ and P2 relatively delayed are shown in Fig 1. The wide range of P1 and M1 stages conditional on P2 at ‘Ci’ is illustrated in Fig 2.

### Odds ratio

The chance of P2 being observed at ‘Cco’ or later stages was (15/0)/(109/89) = 25.3 times greater if P1 was in stage ‘Ri’ or earlier, compared to P1 later than ‘Ri’ (OR 25.34, 95% confidence interval 1.50–429.37, P = 0.0252). The chance of P2 being observed at ‘Cco’ or later stages was (19/0)/(105/89) = 33.1 times greater if M1 was in stage ‘R½’ or earlier, compared to P1 later than ‘R¾’ (OR 33.09, 95% confidence interval 1.97–555.77, P = 0.0151).

Our results show that the age of P2 initiation is highly variable and P2 stage ‘Ci’ can be considerably delayed in timing as well as relative development. Stage ‘Ci’ of P2 can be expressed in chronological age but assessing if a child is dentally advanced or delayed is better expressed in developmental terms. Stage ‘Ci’ of P2 was most frequently seen during the first half of P1 crown formation stages. In dentally advanced children P2 initiates soon after P1 and in dentally delayed children, P2 initiates considerably later. Stage P2 ‘Ci’ was observed one to five stages delayed relative to P1. A one stage difference between P1 and P2 could be considered as a dentally advanced child. A difference of four or five stages between P1 and P2, where P2 ‘Ci’ is observed towards the end of P1 crown formation, could be considered as a dentally delayed child where the sequence of tooth formation has a longer duration. Once a few millimetres of root are visible on P1, agenesis of P2 is likely. Similarly, P2 ‘Ci’ was most frequently seen during late crown stages and early root stages of M1. A dentally advanced child may present with P2 ‘Ci’ prior to completion of M1 crown, while M1 ‘R¼’ or ‘R½’ would be regarded as relatively delayed. Once root length is considerably longer than crown height, agenesis of P2 is likely. These threshold stages of M1 and P1 could be tested on another sample ideally on a longitudinal radiographic collection that includes young children.

The bar charts (Fig 2) are not normally distributed because our radiographic sample did not include one year old children and the two year olds are not uniform for age (of the 21 two year olds, 3 were just two years old, while 18 were at least 2.5). Dental radiographs of very young children are rare and this archive, collected over many years is unique in this regard.

The age of initiation of P2 at 2½ years of age from an early anatomical study is probably based on at most two individuals [11]. Several radiographic studies from birth or of very young children describe the timing of tooth stages. The age of crypt formation of P2 is reported at a mean age of 3.3 years [4]. A radiographic study of children aged 3 to 7 years reports that crypt stage of P2 was evident in 49% of three years olds [12]. The most well-known dental reference data is Moorrees *et al*. [5] and these charts show 95% of children reaching P2 stage ‘Ci’ stage after 2nd and before 4^th^ birthday. Another study from birth reports the median age of attainment of cusp initiation of P2 at 3.32 years in boys and 3.20 years in girls [13]. A recent study with a minimum age of 2.07 years reported mean age of P2 crypt at 2.73 girls 3.00 boys and ‘Ci’ at 3.20 girls 3.55 boys [14]. These studies all report mean age of attainment and this indicates the age when half of children show this stage, in other words half the children initiated P2 prior to this age and half older than mean age. Another study reports that 20% of the crown of P1 was evident when P2 initiated [15].

**Fig 3:**
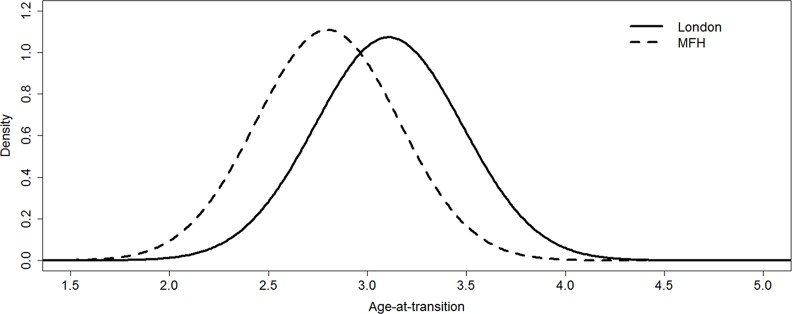
Density plot for transition age P2 stage ‘Ci’. London data from archive, MFH data from Shackelford *et al*. [11].

Fig 3 shows the age of transition from P2 crypt to P2 ‘Ci’ mean age of 3.31 (SD 0.29), calculated from 933 radiographs of children aged 2–8 from the last author’s unpublished data and 78 radiographs of skeletal remains of known age at death aged 1–2 (see AlQahtani *et al*., [16]) illustrated in Fig 3. This approach (probit regression/ transition analysis) is conditional on stage rather than age and the density curve shows the age of transition into P2 ‘Ci’. The dashed line in Fig 3 shows age of transition from Moorrees data (recently tabulated by Shackelford *et al*. [17]) mean age P2 stage ‘Ci’ 2.80 years, SD 0.36).

Late onset of P2 mineralisation could give false-positive diagnosis of agenesis on radiographs [1] and diagnosis of P2 before age 7 is not conclusive [7]. Several reports report late P2 development after 9 or 10 years of age [6, 8, 18]. Relative formation of adjacent teeth can be seen from one of these reports with P1 ‘Ci’ at P1 at ‘R¾’ and M1 ‘Ac’ at 9.5 years [8]. In an ideal world, a study predicting agenesis should utilize longitudinal radiographs of the same children over time, however, dental radiographs are now only taken in the course of diagnosis and treatment. Threshold stages predicting agenesis of P2 could be tested on the valuable, historical longitudinal collections of dental radiographs.

One of the strengths of this study is the age range of the sample, however a limitation is the cross-sectional nature of the radiographs, the non-random selection of radiographs and the lack of full medical history from each patient. A further limitation is the difficulty visualising early premolar tooth stages on dental radiographs. The crypt of P2 is not always clearly defined and a visible cusp tip can be seen prior to the inferior border of the crypt (see Fig 1).

Our results have some clinical importance. A panoramic radiographs prior to eruption of permanent teeth can be of value. Early diagnosis of P2 agenesis can influence a clinical decision whether to save or extract a deciduous second molar or if extracted, whether to maintain or close the space. Early planning minimises the likelihood of a developing malocclusion and will influence the orthodontic treatment plan. Early diagnosis of P2 agenesis also influence the treatment plan for the first permanent molar in an older patient. If P2 cannot be observed once M1 has developed beyond ‘R½’ and P1 beyond ‘Crc’, a heavily restored or carious M1, in a borderline extraction case might be saved, rather than run the risk of two missing units in a single quadrant making subsequent management increasingly complex. Furthermore, the findings can be used to limit emotional and functional problems associated with agenesis of mandibular second premolars and its associated dental anomalies particularly during adolescence, via allowing for early diagnosis and the development of a definitive treatment plan through interdisciplinary management and patient counselling. Treatment can be less complicated and more cost effective as a consequence of early diagnosis and treatment planning, impacting on public health departments and health insurance companies as well as the individual and family levels.

In summary, the probability of observing P2 at ‘Ci’ stage decreases as both the adjacent P1 and M1 mature and their development can help predict the likelihood of mandibular P2 agenesis. By the time M1 had reached stage ‘R¾’ and P1 stage ‘Ri’ no individual in this sample showed evidence of initial cusp formation of P2. These results suggest that once M1 has developed beyond ‘R½’ and P1 ‘Crc’ and P2 is not observed at ‘Ci’ stage, P2 is unlikely to develop.

## Supporting Information

S1 File Contains supporting table of raw data including sex, age and tooth scores of P1, P2 and M1. (PDF)
