# Supplementary material for: Predicting Agenesis of the Mandibular Second Premolar from Adjacent Teeth
Source: PLoS One. 2015 Dec 16;10(12):e0144180. doi: 10.1371/journal.pone.0144180 (PMC4682943; doi:10.1371/journal.pone.0144180)
Supplement: S1 File — (PDF) [file pone.0144180.s001.pdf]

Sex=m male, f female
Age in decimal years
P1=first premolar
P2=second premolar
M1=first molar
Tooth stages:1=Ci, 2=Cco, 3=Coc, 4=C1/2, 5=C3/4, 6=Crc, 7=Ri, 8=R1/4, 9=R1/2, 10=R3/4

| sex | age | P1 | P2 | M1 |
|---|---|---|---|---|
| m | | 4 | 1 | 6 |
| m | | 5 | 1 | 9 |
| m | | 5 | 2 | 7 |
| m | 2.08 | 3 | 1 | 6 |
| m | 2.50 | 2 | 1 | 6 |
| m | 2.53 | 3 | 1 | 6 |
| m | 2.53 | 2 | 1 | 6 |
| m | 2.54 | 3 | 1 | 6 |
| m | 2.59 | 2 | 1 | 6 |
| m | 2.68 | 3 | 1 | 6 |
| m | 2.78 | 3 | 1 | 6 |
| m | 2.81 | 2 | 1 | 7 |
| m | 2.89 | 2 | 1 | 6 |
| m | 2.91 | 2 | 1 | 5 |
| m | 2.92 | 2 | 1 | 6 |
| m | 3.00 | 4 | 3 | 7 |
| m | 3.06 | 2 | 1 | 5 |
| m | 3.13 | 3 | 3 | 6 |
| m | 3.13 | 3 | 2 | 6 |
| m | 3.20 | 4 | 1 | 6 |
| m | 3.24 | 4 | 1 | 6 |
| m | 3.25 | 3 | 2 | 6 |
| m | 3.26 | 5 | 2 | 8 |
| m | 3.39 | 5 | 2 | 5 |
| m | 3.41 | 3 | 2 | 6 |
| m | 3.43 | 5 | 1 | 6 |
| m | 3.44 | 2 | 3 | 6 |
| m | 3.52 | 3 | 1 | 6 |
| m | 3.53 | 2 | 1 | 6 |
| m | 3.58 | 2 | 1 | 6 |
| m | 3.60 | 3 | 1 | 6 |
| m | 3.68 | 4 | 1 | 7 |
| m | 3.70 | 3 | 1 | 6 |
| m | 3.70 | 5 | 2 | 8 |
| m | 3.72 | 4 | 3 | 6 |
| m | 3.74 | 4 | 2 | 6 |
| m | 3.77 | 6 | 2 | 8 |
| m | 3.77 | 3 | 1 | 6 |

| | | | | |
|---|---|---|---|---|
| m | 3.79 | 5 | 1 | 7 |
| m | 3.81 | 3 | 1 | 7 |
| m | 3.82 | 4 | 3 | 8 |
| m | 3.83 | 3 | 1 | 6 |
| m | 3.84 | 2 | 1 | 6 |
| m | 3.84 | 5 | 2 | 7 |
| m | 3.85 | 4 | 1 | 6 |
| m | 3.85 | 5 | 2 | 6 |
| m | 3.86 | 3 | 2 | 7 |
| m | 3.86 | 5 | 2 | 8 |
| m | 3.87 | 5 | 3 | 5 |
| m | 3.87 | 5 | 2 | 6 |
| m | 3.87 | 3 | 1 | 6 |
| m | 3.87 | 3 | 1 | 6 |
| m | 3.87 | 5 | 1 | 6 |
| m | 3.87 | 4 | 2 | 8 |
| m | 3.88 | 4 | 1 | 6 |
| m | 3.88 | 5 | 2 | 7 |
| m | 3.89 | 3 | 1 | 8 |
| m | 3.90 | 5 | 3 | 8 |
| m | 3.90 | 5 | 3 | 8 |
| m | 3.92 | 4 | 3 | 5 |
| m | 3.93 | 5 | 2 | 5 |
| m | 3.93 | 4 | 2 | 7 |
| m | 3.97 | 4 | 1 | 7 |
| m | 3.97 | 4 | 3 | 8 |
| m | 3.66 | 3 | 1 | 6 |
| m | 4.00 | 5 | 2 | 7 |
| m | 4.01 | 4 | 3 | 6 |
| m | 4.02 | 3 | 1 | 7 |
| m | 4.02 | 4 | 1 | 8 |
| m | 4.03 | 4 | 1 | 6 |
| m | 4.03 | 3 | 2 | 7 |
| m | 4.03 | 3 | 1 | 8 |
| m | 4.10 | 5 | 3 | 7 |
| m | 4.13 | 4 | 3 | 7 |
| m | 4.16 | 3 | 2 | 6 |
| m | 4.18 | 3 | 1 | 6 |
| m | 4.18 | 3 | 1 | 8 |
| m | 4.20 | 2 | 1 | 8 |
| m | 4.26 | 4 | 1 | 7 |
| m | 4.28 | 5 | 3 | 8 |
| m | 4.32 | 4 | 1 | 7 |
| m | 4.32 | 5 | 3 | 8 |
| m | 4.33 | 5 | 2 | 6 |
| m | 4.33 | 5 | 2 | 8 |

| | | | | |
|---|---|---|---|---|
| m | 4.35 | 5 | 3 | 7 |
| m | 4.37 | 5 | 3 | 7 |
| m | 4.39 | 5 | 3 | 8 |
| m | 4.45 | 5 | 2 | 7 |
| m | 4.48 | 4 | 3 | 7 |
| m | 4.55 | 3 | 1 | 8 |
| m | 4.61 | 5 | 3 | 8 |
| m | 4.62 | 6 | 4 | 10 |
| m | 4.62 | 5 | 2 | 7 |
| m | 4.68 | 5 | 4 | 5 |
| m | 4.73 | 4 | 2 | 6 |
| m | 4.74 | 3 | 2 | 8 |
| m | 4.86 | 4 | 1 | 8 |
| m | 4.87 | 4 | 2 | 7 |
| m | 4.90 | 5 | 2 | 8 |
| m | 4.92 | 7 | 5 | 9 |
| m | 4.97 | 7 | 4 | 8 |
| m | 5.00 | 4 | 2 | 8 |
| m | 6.24 | 7 | 6 | 10 |
| m | 6.25 | 7 | 6 | 10 |
| m | 6.37 | 8 | 6 | 10 |
| m | 6.44 | 6 | 5 | 10 |
| m | 6.64 | 3 | 1 | 7 |
| m | 6.74 | 7 | 6 | 10 |
| m | 6.97 | 7 | 6 | 10 |
| m | 7.01 | 7 | 5 | 10 |
| m | 7.15 | 7 | 5 | 10 |
| m | 7.51 | 7 | 6 | 10 |
| m | 8.66 | 3 | 2 | 7 |
| f | | 4 | 2 | 8 |
| f | 2.47 | 3 | 1 | 5 |
| f | 2.47 | 2 | 1 | 6 |
| f | 2.57 | 3 | 1 | 6 |
| f | 2.59 | 5 | 2 | 6 |
| f | 2.60 | 3 | 1 | 6 |
| f | 2.74 | 3 | 1 | 6 |
| f | 2.75 | 4 | 3 | 8 |
| f | 2.77 | 3 | 1 | 6 |
| f | 2.98 | 4 | 1 | 8 |
| f | 3.00 | 4 | 2 | 9 |
| f | 3.07 | 4 | 1 | 7 |
| f | 3.11 | 5 | 3 | 7 |
| f | 3.18 | 5 | 3 | 6 |
| f | 3.19 | 2 | 1 | 6 |
| f | 3.22 | 3 | 1 | 6 |
| f | 3.33 | 3 | 2 | 5 |

| | | | | |
|---|---|---|---|---|
| f | 3.33 | 4 | 1 | 6 |
| f | 3.33 | 3 | 1 | 7 |
| f | 3.35 | 2 | 1 | 6 |
| f | 3.36 | 4 | 1 | 8 |
| f | 3.37 | 2 | 1 | 6 |
| f | 3.37 | 2 | 1 | 6 |
| f | 3.37 | 5 | 1 | 6 |
| f | 3.38 | 4 | 1 | 7 |
| f | 3.39 | 4 | 2 | 6 |
| f | 3.41 | 4 | 1 | 6 |
| f | 3.41 | 4 | 1 | 7 |
| f | 3.46 | 2 | 1 | 6 |
| f | 3.49 | 3 | 2 | 8 |
| f | 3.58 | 2 | 1 | 6 |
| f | 3.58 | 3 | 1 | 6 |
| f | 3.60 | 4 | 3 | 6 |
| f | 3.65 | 5 | 2 | 6 |
| f | 3.67 | 4 | 2 | 6 |
| f | 3.67 | 4 | 1 | 6 |
| f | 3.67 | 4 | 2 | 9 |
| f | 3.68 | 4 | 2 | 8 |
| f | 3.70 | 5 | 2 | 7 |
| f | 3.70 | 2 | 1 | 8 |
| f | 3.71 | 4 | 1 | 6 |
| f | 3.72 | 5 | 3 | 8 |
| f | 3.73 | 3 | 1 | 7 |
| f | 3.75 | 3 | 1 | 7 |
| f | 3.48 | 5 | 1 | 7 |
| f | 3.77 | 4 | 2 | 8 |
| f | 3.80 | 3 | 1 | 6 |
| f | 3.80 | 3 | 1 | 8 |
| f | 3.82 | 4 | 2 | 6 |
| f | 3.85 | 4 | 3 | 7 |
| f | 3.91 | 3 | 1 | 7 |
| f | 3.96 | 2 | 1 | 6 |
| f | 3.99 | 5 | 2 | 6 |
| f | 4.00 | 4 | 2 | 7 |
| f | 4.00 | 4 | 2 | 8 |
| f | 4.00 | 4 | 3 | 9 |
| f | 4.02 | 5 | 3 | 6 |
| f | 4.02 | 5 | 1 | 8 |
| f | 4.03 | 6 | 1 | 8 |
| f | 4.04 | 3 | 2 | 6 |
| f | 4.05 | 6 | 3 | 8 |
| f | 4.09 | 3 | 1 | 6 |
| f | 4.11 | 5 | 1 | 9 |

| | | | | |
|---|---|---|---|---|
| f | 4.21 | 5 | 3 | 9 |
| f | 4.25 | 4 | 2 | 8 |
| f | 4.26 | 4 | 1 | 8 |
| f | 4.27 | 4 | 2 | 7 |
| f | 4.30 | 4 | 2 | 7 |
| f | 4.31 | 5 | 2 | 8 |
| f | 4.32 | 5 | 3 | 8 |
| f | 4.35 | 5 | 2 | 6 |
| f | 4.36 | 4 | 1 | 6 |
| f | 4.37 | 5 | 2 | 8 |
| f | 4.40 | 5 | 3 | 8 |
| f | 4.41 | 5 | 3 | 7 |
| f | 4.43 | 5 | 2 | 7 |
| f | 4.56 | 4 | 2 | 8 |
| f | 4.57 | 5 | 4 | 8 |
| f | 4.62 | 7 | 5 | 10 |
| f | 4.64 | 5 | 1 | 5 |
| f | 4.76 | 5 | 3 | 8 |
| f | 4.77 | 5 | 2 | 6 |
| f | 4.84 | 5 | 4 | 8 |
| f | 4.91 | 6 | 4 | 10 |
| f | 5.00 | 6 | 3 | 8 |
| f | 5.44 | 5 | 2 | 9 |
| f | 5.53 | 5 | 2 | 9 |
| f | 5.59 | 6 | 4 | 8 |
| f | 6.00 | 7 | 6 | 10 |
| f | 6.01 | 8 | 6 | 10 |
| f | 6.23 | 6 | 5 | 10 |
| f | 6.41 | 7 | 6 | 10 |
| f | 6.70 | 7 | 6 | 10 |
| f | 3.98 | 5 | 3 | 8 |
| f | 7.05 | 6 | 5 | 10 |
| f | 7.66 | 4 | 2 | 7 |
| f | 8.31 | 5 | 4 | 10 |
| f | 9.34 | 3 | 2 | 8 |
| f | 9.42 | 4 | 2 | 7 |
| f | 9.56 | 5 | 2 | 6 |
